# Incidence Prediction for the 2017-2018 Influenza Season in the United States with an Evolution-informed Model

**DOI:** 10.1371/currents.outbreaks.6f03b36587ae74b11353c1127cbe7d0e

**Published:** 2018-01-17

**Authors:** Xiangjun Du, Mercedes Pascual

**Affiliations:** Ecology and Evolution, University of Chicago, Chicago, Illinois, USA; Ecology and Evolution, University of Chicago, Chicago, Illinois, USA

**Keywords:** 2017-2018, evolution, forecast, incidence, Influenza, model, seasonal, United States

## Abstract

**Introduction:**

Seasonal influenza is responsible for a high disease burden in the United States and worldwide. Predicting outbreak size in advance can contribute to the timely control of seasonal influenza by informing health care and vaccination planning.

**Methods:**

Recently, a process-based model was developed for forecasting incidence dynamics ahead of the season, with the approach validated by several statistical criteria, including an accurate real-time prediction for the past 2016-2017 influenza season before it started.

**Results:**

Based on this model and data up to June 2017, a forecast for the upcoming 2017-2018 influenza season is presented here, indicating an above-average, moderately severe, outbreak dominated by the H3N2 subtype.

**Discussion:**

The prediction is consistent with surveillance data so far, which already indicate the predominance of H3N2. The forecast for the upcoming 2017-2018 influenza season reinforces the importance of the on-going vaccination campaign.

## Incidence prediction for the 2017-2018 influenza season in the United States

Human infections caused by the seasonal influenza virus impose a large burden on public health in the United States and worldwide. Advanced forecasts of the severity of the upcoming influenza season can contribute to timely preparation for the season, including resource allocation and vaccination campaigns. Existing computational methods have already been developed for this purpose, which can be largely classified into two main classes: the first one focuses on within-season forecasting and relies on updated incidence information as the season develops^1^; the second generates a prediction for the next season based on the data of the current season^2^^,^^3^. Whereas the former predicts absolute severity information and peak timing within season (weeks), the latter produces a forecast with a longer lead time for the next season, but is most accurate for the relative frequency of different lineages (or antigenic clusters) and not for the absolute severity or incidence.

Recently, a process-based model (EvoEpiFlu) that incorporates evolutionary information into a modified SIRS system of equations (for Susceptible, Infected, Recovered and Susceptible immune classes in the population) was developed to predict absolute influenza incidence for subtype H3N2 for the United States^4^. EvoEpiFlu makes use of evolutionary information related to antigenic change based only on sequences to infer H3N2 incidence from the summer to the upcoming influenza season. Two formulations of EvoEpiFlu were implemented: as its name indicates, the ‘continuous’ version relies on evolutionary change information continuously in time via an evolutionary index constructed from changes in amino acid sequences for epitope sites in the antigen protein HA between current and past strains, with the temporal scale of the past estimated during model optimization (Figure 1). The second formulation or ‘cluster’ model takes advantage of the notion of antigenic cluster transitions via a genotype-phenotype map^5^ which provides the proportion of antigenic variants (PAV) (Figure 1). PAV is also calculated based on readily available sequences^4^ and its value is high when there is an antigenic cluster replacement or transition. Based only on the sequences and the previous incidence surveillance, EvoEpiFlu produces skillful forecasts one season ahead (during the summer with data up to June) for both the severity of total incidence caused by H3N2 in the upcoming season and whether subtype H3N2 will be dominant^4^. EvoEpiFlu was validated by leave-one-out cross-validation for the period between 2003 and 2011, and with one-season-ahead forecasts for the period between 2011 and 2017 (with significant respective correlations of 0.88 and 0.90 between observed and predicted seasonal total cases) (Figure 1B. The prediction for the 2016-2017 influenza season was a real-time forecast because it was produced before the season arrived and validated only recently with the updated surveillance data. It predicted a high H3N2 season with an estimated median incidence rate (proportion of the population infected) of 0.11 ([0.07, 0.15], 95% confidence interval), consistent with the later observation of a high season with an incidence rate of 0.10^4^.

**Model and Forecasts.**
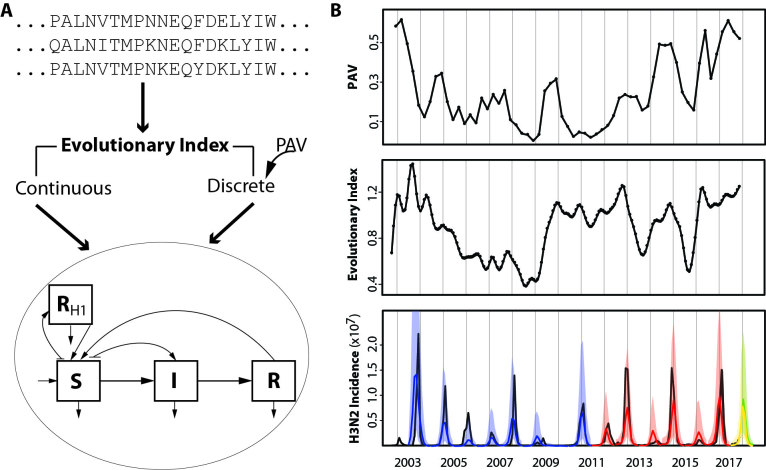
**(A)** Workflow of the EvoEpiFlu model. The evolutionary index is calculated as a weighted sum of sequence distances (based on epitope sites of HA) to compare strains each month to strains in the past, with distances weighted by a decay function back in time. Another approach relies on a discrete version of evolutionary change and is based on a previously-published genotype-phenotype map^5^ , producing the proportion of antigenic variants (PAV) over time. In order to make the forecast, two versions of the model were developed that incorporate the evolutionary index either in continuous or discrete form (in the continuous model and discrete model respectively). These quantities are included as covariates in a modified SIRS formulation for the transmission dynamics of the disease (tracking three main classes of individuals, S for susceptible, I for infected and infectious, and R for recovered and immune, as well as an additional class for immune to H1N1, which is informed by observed H1N1 incidence). In the models, evolutionary change determines the rate of return of immune individuals to the susceptible class, mimicking a loss of protection as the virus evolves. **(B)** Model evolutionary components and forecasts (based on available data up to November, 2017 at the time of publication). Top panel: PAV calculated for strains in each quarter compared to strains in the past 12 months. Middle panel: the evolutionary index calculated monthly. Bottom panel: the H3N2 incidence forecast based on data up to June, 2017 for the upcoming 2017-2018 season in the US based on the cluster version of EvoEpiFlu is shown in yellow. An updated forecast using data up to November 2017 is also shown in green. The observed data are indicated in black, with observed data for the 2017-2018 season up to December at the time of publication shown with the dotted line; forecasts are color-coded with their corresponding 95% uncertainty intervals, with blue for leave-one-out cross-validation, red for one-season-ahead forecasting (for out-of-fit data), and for the real-time forecast of the 2016-2017 season. For model details see^4^ , from which the forecasts up to 2016-2017 are taken.

Encouraged by the prediction skill of EvoEpiFlu, we produced a forecast for the upcoming 2017-2018 season. Both the PAV and the Evolution Index are high (Figure 1B, top and middle panels), indicating that a new antigenic cluster is likely to be circulating in the upcoming season with a significant evolutionary change in antigenic space. On this basis and the data up to June 2017, the EvoEpiFlu’s cluster model predicts that the upcoming 2017-2018 season will be H3N2-dominant (Figure 1B, bottom panel, yellow line), of moderate severity for the United States with an estimated median incidence rate of 0.09 ([0.05,0.14], 95% confidence interval) compared to an average incidence rate of 0.05 for H3N2 in the past fifteen years. The forecast based on the most recently available data (up to November 2017) gives similar results (Figure 1B, bottom panel, green line).

The weekly influenza report by the CDC, FluView, for the past week (week 48, ending on December 2, 2017) and data accumulated from the start of the season (week 40, October 1, 2017) suggest that H3N2 already predominates (74% and 77% respectively). Consistent with the study by Goldstein showing that early circulation of one influenza subtype is associated with a reduced incidence of other subtypes^6^, recent data between 2003-2017 suggests that there is a high probability that H3N2 will be dominant in the following winter season (8 out of 9 seasons) when H3N2 already predominates in late summer (weeks 30-39). Based on surveillance, H3N2 has also been dominant in many countries in the Southern Hemisphere although a dominant subclade with substantial antigenic drift has yet to emerge^7^. These observations suggest a high likelihood of H3N2 spreading to regions in the Northern Hemisphere, including the United States and Europe.

The prediction of H3N2-dominance for the 2017-2018 season if confirmed would result into two continuous seasons dominated by the same subtype H3N2, an unusual pattern that only happened three times in the past twenty years. In the context of the low vaccination rate last year (47%), the prediction for the 2017-2018 season by EvoEpiFlu should provide additional information and an early-warning for people to get vaccinated.

## Competing Interests

We declare we have no conflict of interests.

## Data Availability Statement

All the data used in this study are from the public database and are publicly available to other researchers.

## Corresponding Author

Xiangjun Du, xiangjundu@gmail.com
